# Prevalence, Associated Factors, and Temporal Trends of *Brucella* Detection Across Human and Animal Hosts in Bangladesh: A 25‐Year Meta‐Analysis

**DOI:** 10.1002/vms3.71005

**Published:** 2026-05-28

**Authors:** Radwan Raquib, Farhaj Ahammed Arnob, Riyadh Hossain, Ahsan Raquib, Tajul Islam Mamun

**Affiliations:** ^1^ Faculty of Veterinary Animal and Biomedical Sciences Sylhet Agricultural University Sylhet Bangladesh; ^2^ Department of Parasitology Sylhet Agricultural University Sylhet Bangladesh; ^3^ Department of Statistics Noakhali Science and Technology University Noakhali Bangladesh; ^4^ Department of Epidemiology and Public Health Sylhet Agricultural University Sylhet Bangladesh

**Keywords:** associated factors, Bangladesh, *Brucella*, disease trends, meta‐analysis, prevalence, zoonotic disease

## Abstract

**Background:**

Brucellosis remains an important zoonotic disease in Bangladesh, yet evidence on its prevalence across human and animal hosts is fragmented and heterogeneous.

**Objectives:**

This study aims to synthesize evidence on *Brucella* detection across human and animal hosts in Bangladesh and to evaluate associated study‐level factors and temporal patterns.

**Methods:**

The data were extracted from eligible studies published between 2000 and 2024 following a systematic review framework. Pooled prevalence was estimated using a random‐effects meta‐analysis. Heterogeneity was assessed using Cochran's *Q* and *I* ^2^ statistics. Subgroup meta‐analyses and univariable meta‐regression were conducted to examine variation by host species, diagnostic method, and study‐level factors. Forecasting was performed using ARIMA models on annual study‐reported *Brucella* detection, with model performance evaluated using AIC, MAE, and RMSE.

**Results:**

The pooled *Brucella* detection across hosts was 3.75% (95% CI: 3.07–4.56), with substantial heterogeneity (*I* ^2^ = 89.5%). Detection was the highest in dogs (7.49%) and the lowest in horses (1.78%)). Among evaluated variables, abortion history was the only study‐level factorsignificantly associated with higher *Brucella* positivity, with aborted animals showing approximately 10 times higher odds of detection compared to non‐aborted animals (OR = 10.09, 95% CI: 4.64–21.93, *p* <0.001). Temporal patterns suggested a decreasing trend in goats and an increasing trend in cattle; however, these likely reflect variation in study design, diagnostics, and coverage rather than true epidemiological change.

**Conclusion:**

*Brucella* exposure and detection persist across multiple host species in Bangladesh, with variation largely driven by methodological and host‐related differences. These findings underscore the need for standardized diagnostic and reporting approaches and support integrated One Health surveillance to improve interpretation and control strategies in Bangladesh.

## Graphical abstract

This systematic review and meta‐analysis synthesizes *Brucella* prevalence across human and animal hosts in Bangladesh. Dogs showed the highest prevalence (7.49%), followed by goats (3.86%), cattle (3.67%), humans (2.94%), and horses (1.78%). Abortion history was significantly associated with *Brucella* infection, underscoring the need for integrated One Health surveillance.

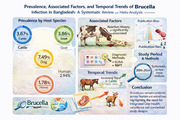

## 1 Introduction

Brucellosis is one of the major global zoonotic diseases, caused by Gram‐negative bacteria of the genus *Brucella*, and is responsible for substantial economic losses in the livestock industry worldwide (Rossetti et al. 2017; SciELO—Public Health 2025). *Brucella* infects almost half a million humans worldwide each year (Seleem et al. 2010; Pappas et al. 2006). However, it is recognized as a neglected zoonosis by the World Health Organization (Franc et al. 2018) and the World Organization for Animal Health (Zhang et al. 2019) because the health system does not prioritize this disease. It mainly invades the reproductive tract of domestic animals and can be transmitted vertically and horizontally in them (Moreno 2014; Tadesse 2016). Contact with infected animals and drinking milk without pasteurization can cause the transmission of this pathogen to humans (Moreno 2014). Pet animals, such as dogs, can be infected by ingesting vaginal discharge from diseased animals (M. A. Islam et al. 2013).

Brucellosis is endemic in Bangladesh, as in some other developing countries, including Africa (Maurice et al. 2013; Ducrotoy et al. 2017; Vhoko et al. 2018), Central America (Moreno 2002), Latin America (Lucero et al. 2008), and the Middle East (Refai 2002; Musallam et al. 2016). Clinical pictures of brucellosis vary widely among humans and animals. In animals, clinical signs are abortion, stillbirth, low survival rate of calves, delayed calving, reduced milk production, and infertility in male animals (Khan and Zahoor 2018). In humans, symptoms are weakness, pain in joints and muscles, enlargement of the liver and spleen, headache, and undulant fever, accompanied by a negligible death rate, with the infection lasting for several years (Madkour 2001). Humans with brucellosis most often have a fever that is mistaken for malaria, which is another endemic disease in Bangladesh (M. J. Corbel 2006).

Bangladesh is a South Asian country with a fast‐growing livestock industry where almost every family has domestic or pet animals in their houses. They hold a close relationship with them, particularly with sheep and goats, which is the principal source of human brucellosis (Moreno 2014), although humans can acquire this pathogen from a wide range of animal species. Existing studies on the prevalence of human brucellosis showed remarkable differences in the approximate range from 2.51% in Kathmandu, Nepal (Pokhrel et al. 2021), to 44.7% in the Sylhet district of Bangladesh (Akhtar et al. 2020). A meta‐analysis between 2008 and 2017 with 22 studies on brucellosis found 17%, 2%, 33%, and 3% prevalence of brucellosis in bovine, caprine, porcine, and sheep‐goat, respectively, in the northern region of India (Barman et al. 2020). These findings may be relevant to Bangladesh due to its geographical proximity and nearly similar socio‐economic settings, but still not enough for policymakers to make control strategies.

For accurate estimation, a systematic evaluation of available studies is necessary, as individual studies cannot reliably represent the overall magnitude of a disease across a broader geographic area. Despite the endemicity of brucellosis in Bangladesh, comprehensive, nationally synthesized evidence on *Brucella* detection across human and animal hosts remains limited. In particular, variation in reported prevalence by host species, demographic characteristics, and diagnostic methods has not been systematically evaluated. Therefore, the objective of this study was to synthesize available evidence on *Brucella* detection across human and animal hosts in Bangladesh and to examine associated study‐level factors and temporal patterns.

## 2 Methods

### 2.1 Systematic Review and Meta‐Analysis

#### 2.1.1 Study Design and Search Strategy

This study employed a systematic review and meta‐analysis to synthesize existing articles on the evidence of *Brucella* detection across human and animal hosts in Bangladesh and to examine associated study‐level factors and temporal patterns. The review was conducted in accordance with the Preferred Reporting Items for Systematic Reviews and Meta‐Analyses (PRISMA) guidelines to ensure transparency and reproducibility (Moher et al. 2015) (Table S1). A comprehensive literature search was conducted using bibliographic databases, including PubMed, and Scopus supplemented by targeted searches in Google Scholar, which served as a search engine to identify additional relevant evidence. The search covered predefined combinations of keywords such as ‘*Brucella*’, ‘Brucella detection’, ‘prevalence’, ‘seroprevalence’, ‘risk factors’, and ‘Bangladesh’ (Table 1), adapted from previously published systematic reviews (Musallam et al. 2016; Mamun et al. 2025; H. Talukder et al. 2023; Raquib et al. 2022, 2025). The study protocol was registered in the PROSPERO database (ID: CRD420251082670).

**TABLE 1:** Algorithm for study search to identify published articles on prevalence of brucellosis in humans and animals.

Search function	Search term
Any of	Brucellosis OR Malta fever OR *Brucella* OR ‘*Brucella melitensis*’ OR ‘*Brucella abortus*’
AND Any of	Prevalence OR Occurrence OR Investigation OR Detection OR Identification OR Characterization OR Investigation
AND Any of	Cattle OR Goat OR Sheep OR Buffalo OR Horse OR Human OR Dog OR Pig OR animals
Location	Bangladesh
Period	2000–2024

#### 2.1.2 Eligibility Criteria

Eligible studies were included if they reported the prevalence of *Brucella* detection in human or animal species in Bangladesh and provided sufficient extractable data using recognized diagnostic methods, including the Rose Bengal Test (RBT), enzyme‐linked immunosorbent assay (ELISA), and polymerase chain reaction (PCR). These methods capture different aspects of *Brucella* detection: RBT and ELISA are widely used serological screening tools for population‐level surveillance, whereas PCR enables direct detection of *Brucella* DNA and is considered a confirmatory method for identifying active infection (Dadar et al. 2026).

Included studies were cross‐sectional or case‐control observational studies published in English between January 2000 and December 2024 and available in full text. Grey literature (e.g., government reports and academic theses) was reviewed to assess the completeness of the evidence but was excluded from the quantitative meta‐analysis due to the lack of peer review. Studies with insufficient or non‐extractable data, as well as those from non‐peer‐reviewed sources, were excluded. Review articles, conference proceedings, case reports, editorials, theses, and opinion papers were also excluded. Duplicate publications and studies conducted outside Bangladesh were removed. Study selection was performed independently by two authors, with disagreements resolved through discussion with a third author. The reference lists of the included studies were manually screened to identify additional relevant articles.

#### 2.1.3 Data Extraction

Data extraction was conducted systematically using a predefined data extraction form to ensure consistency and accuracy. Information was extracted independently from each eligible study and recorded in a Microsoft Excel spreadsheet. Extracted variables included the first author's name, year of publication, study location (district), study design, host species (e.g., human, cattle, goat), sample size, and reported prevalence or seroprevalence of *Brucella* detection. The details of diagnostic methods used, including PCR, ELISA, RBT, standard agglutination test (SAT), and milk ring test (MRT), were recorded. When multiple diagnostic tests were reported within a single study, the test with the highest reported diagnostic performance was selected for prevalence extraction based on the following predefined hierarchy: RT‐PCR > conventional PCR > ELISA > RBT > SAT > MRT, consistent with previous studies (Asaad and Alqahtani 2012; Getachew et al. 2016; Sadhu et al. 2015; Sharma et al. 2016). Additional extracted variables included sex, breed, abortion history, and other study‐reported factors examined in relation to *Brucella* detection. These variables were treated as study‐level associated factors. Information on sampling and data collection methods was also recorded to support assessment of study quality and comparability.

#### 2.1.4 Quality Assessment of Selected Studies

Assessment and assurance of the quality of the selected studies were done through a checklist devised by the Joanna Briggs Institute (JBI) for studies reporting prevalence data (JBI 2020). A scoring approach was employed where one point was allotted for the presence of each of the following items in the selected article: study objective, sampling area, study period, sample close representation of the general population, random sampling, sample size calculation, diagnostic techniques used, correct analysis, categorized by sex, and consistent methodology.

*Case definition*: *Brucella* positivity was defined based on detection of *Brucella* exposure or presence using serological (e.g., ELISA, RBT) or molecular (PCR) diagnostic methods as reported in the original studies. Serological methods were interpreted as evidence of prior exposure, whereas PCR‐based methods were considered indicative of current infection. Clinical diagnosis of brucellosis was not required for inclusion, and therefore pooled estimates reflect laboratory/kit‐based evidence of exposure or detection rather than clinically confirmed disease.

#### 2.1.5 Data Analysis

The meta, metafor, and dmetar packages in R (version 4.4.2) were used to estimate pooled *Brucella* detection. All prevalence estimates were logit‐transformed before meta‐analysis to stabilize variances.After analysis, results are back‐transformed. A random‐effects meta‐analysis was used as the primary analytical approach to account for between‐study variability. Statistical heterogeneity among studies was assessed using Cochran's *Q* test and quantified using the *I* ^2^ statistic, which represents the proportion of total variability attributable to between‐study heterogeneity rather than sampling error (Higgins 2003). Values of *I* ^2^ greater than 50% were interpreted as indicating substantial heterogeneity. Given the expected methodological diversity across studies, random‐effects models were applied throughout.

Forest plots were used to visually present pooled prevalence estimates and confidence intervals (Barendregt et al. 2013; Li et al. 2020). Potential small‐study effects were assessed using funnel plots (Figure 3) and Egger's regression test (Table 2) (Egger et al. 1997). Sensitivity analyses, including leave‐one‐out analysis and trim‐and‐fill procedures, were conducted to evaluate the robustness of the pooled estimates. To explore potential sources of heterogeneity, subgroup meta‐analyses were performed by host species (cattle, buffalo, sheep, goat, horse, pig, dog, and human), diagnostic method (PCR, ELISA, RBT, SAT, and others), geographic location, and sex. Additional subgroup analyses for animal studies considered breed (local, exotic, crossbred), abortion history (yes/no), and pregnancy status (yes/no), where data were available. To further examine study‐level associations with reported prevalence, univariable meta‐regression analyses were conducted. Univariable meta‐regression was performed using a generalized linear mixed‐effects model with a logit‐transformed prevalence as the effect size. Between‐study heterogeneity was estimated using maximum likelihood, and the results were expressed as odds ratios (ORs) with 95% confidence intervals.

#### 2.1.6 Forecast Analysis

Exploratory time‐series analyses were conducted using autoregressive integrated moving average (ARIMA) models applied to annual study‐reported prevalence estimates. These analyses were performed solely to illustrate patterns in published data over time and do not represent population‐level surveillance or disease forecasts. Given heterogeneity in study design, diagnostics, and sampling frames, the results were interpreted cautiously as descriptive trend projections. The Box‐Jenkins approach, using autoregressive moving average ARMA or ARIMA models in time‐series analysis, was used to determine whether the time series fits its historical values in order to provide forecasts. Two evaluation measures have been used in this study, which are mean absolute error (MAE) and root mean square error (RMSE), to assess the efficacy of the model. The forecasting technique used averages to analyze several functions to distinguish and predict values. The remaining terms in equations (1) and (2) were used to describe these discrepancies between prognostic values and practical values (Evaluating forecast accuracy (MAE, RMSE, MAPE) 2025).

(1) $$\mathrm{MAE}=\frac{1}{n}\sum_{t=1}^{n}\left|Y_{t}-{\hat{Y}}_{t}\right|,$$

(2) $$\mathrm{MSE}=\frac{1}{\;n}\sum_{t=1}^{n}{\left(Y_{t}-{\hat{Y}}_{t}\right)}^{2}.$$

The final models ARIMA (1,0,1) for goat and cattle, respectively, were selected based on the lowest Akaike Information Criterion (AIC) value and the highest *R* ^2^ value, as shown in Table 3. Exploratory ARIMA models suggested divergent patterns in reported *Brucella* detection for goats and cattle; however, substantial volatility in predicted values indicates sensitivity to heterogeneous input data and limits epidemiological interpretation.

**TABLE 2:** Meta‐regression of *Brucella* association in different categories.

Variables	Category	Univariate meta‐regression		*p* value (between groups)
		Co‐efficient (95% CI)	*p* value (within group)	
Species	Horse	Reference		0.847
	Goat	2.33 (0.26−20.36)	0.441	
	Cattle	2.36 (0.28−19.87)	0.426	
	Buffalo	3.49 (0.37−32.47)	0.271	
	Sheep	2.60 (0.29−23.11)	0.388	
	Human	2.02 (0.22−18.13)	0.529	
	Dog	4.76 (0.38−59.36)	0.224	
	Pig	4.24 (0.27−64.34)	0.290	
Sex	Male	Reference		0.642
	Female	1.13 (0.66–1.91)	0.642	
Breed	Local	Ref		0.566
	Exotic	0.79 (0.24–2.57)	0.702	
	Cross	1.28 (0.70–2.33)	0.409	
Test	TAT	Reference		0.836
	RBT	1.55 (0.59−4.04)	0.363	
	MRT	0.89 (0.27−2.87)	0.850	
	SAT	1.26 (0.44−3.59)	0.657	
	*Brucella* antigen kit	1.99 (0.50−7.83)	0.320	
	ELISA	1.30 (0.49−3.45)	0.593	
	Conventional PCR	1.10 (0.28−4.26)	0.889	
	RT‐PCR	1.36 (0.41−4.50)	0.606	
Sample	Milk	Reference		0.851
	Blood	0.92 (0.43−1.98)	0.851	
Abortion status	No abortion	Reference		<0.001
	Abortion	10.09 (4.64−21.93)	<0.001	
Physiological status	Non‐pregnant	Reference		0.037
	Pregnant	1.71 (1.03−2.84)	0.037	

**TABLE 3:** Model selection for goat and cattle.

Species	Models	AIC	*R* ^2^
Goat	ARIMA (0,0,0)	13.56176	0.237
	ARIMA (1,0,1)	13.25648	0.79658
	ARIMA (2,0,1)	13.38668	0.56725
Cattle	ARIMA (1,0,1)	9.28	0.979
	ARIMA (1,1,2)	9.76	0.245
	ARIMA (2,1,2)	9.83	0.169

## 3 Results

### 3.1 Characteristics of the Included Studies

**FIGURE 1:**
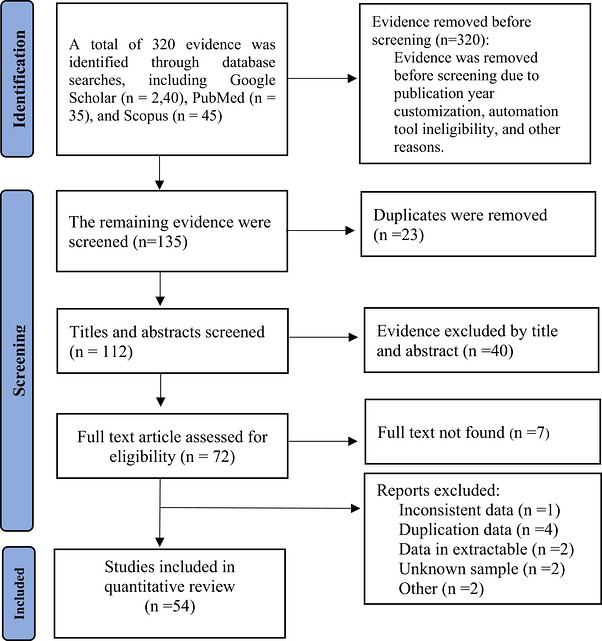
PRISMA flow diagram of literature search and study selection (Moher et al. 2015).

The systematic literature search identified a total of 320 articles. After removal of duplicates and initial screening, 112 studies were assessed based on titles and abstracts, followed by full‐text evaluation of potentially eligible studies. Ultimately, 54 studies met the inclusion criteria and were included in the meta‐analysis, as summarized in the PRISMA flow diagram (Figure 1). In 54 identified studies, a total of 24,966 samples were identified. The highest number of articles (*N* = 32) had prevalence data about cattle (M. S. Ahasan et al. 2010; Maruf et al. 2019; M.K. M.A Rahman et al. 2019; Md. S. Ahasan et al. 2017; M.K.M.A. Rahman et al. 2017; Md. S. Rahman 2012; S. Sikder et al. 2012; M. A. S. Sarker et al. 2018; Sarker, Begum, et al. 2017; Faruque et al. 2021; M. S. Islam et al. 2018; Md. S. Rahman et al. 2013; K. M. R. Amin et al. 2005;M.S Rahman et al. 2006; MAS et al. 2014; M. Rahnan et al. 2019; M. Rahman et al. 2009; M. Rahman et al. 2011; M. Rahman et al. 2010; M. Islam et al. 2007; M. M. Rahman et al. 2020; Nahar and Ahmed 1970; Deb Nath et al. 2023; Dey et al. 2014; K. Amin et al. 2004; Hassan et al. 2014; Sarker, Sarker, et al. 2017; S. Islam et al. 2021; Belal and Ansari 2013; M. A. Islam et al. 2014; Md. S. Rahman et al. 2012; M. S. Rahman et al. 2014) with the highest number of samples (*n* = 13,343). Twelve, 10, and 6 studies were found to have prevalence estimation of brucellosis in goats (Md. S. Ahasan et al. 2017; Md. S. Rahman 2012; M. Rahman et al. 2011; M. K. M. A. Rahman et al. 2013; Md. S. Rahman et al. 2011; Uddin et al. 2007; M.S. Rahman et al. 2013; M. Islam et al. 2012; Shafy et al. 2017; Mollah 2019; Akhter et al. 2014; Munsi et al. 2021), sheep (Md. S. Rahman 2012; M. Rahman et al. 2011; M. K. M. A. Rahman et al. 2013; Md. S. Rahman et al. 2011; Shafy et al. 2017; Akhter et al. 2014; M. Rahman et al. 2024; M. Ahsan et al. 2014; M. Rahman et al. 2013; Gani et al. 2016), and buffalo (Md. S. Rahman 2012; M. Rahman et al. 2011; M. A. S. Sarker, et al. 2017; M. A. Islam et al. 2014; M. S. Rahman et al. 2014; M. Rahman et al. 2014), respectively, brucellosis of horses (Millat et al. 2018) and pig (M. S. Rahman et al. 2013) was identified in one study each, and two studies had prevalence data about dog (B. Talukder et al. 2012; M.S. Rahman et al. 2015). Only eight studies reported human brucellosis, comprising a total sample size of 2982 individuals (A. K. M. A. Rahman et al. 2017; M. Rahnan et al. 2019; M. M. Rahman et al. 2020; Nahar and Ahmed 1970; Akhtar et al. 2020; Rasheduzzaman et al. 2020; A. K. M. A. Rahman et al. 2012; A. A. Rahman et al. 2016). The majority of included studies (*n* = 48) were published between 2010 and 2019, whereas only six studies were published between 2000 and 2009.

### 3.2 *Brucella* Detection in Humans and Animals

Subgroup analyses were conducted by host species, sex, breed, diagnostic method, abortion history, and pregnancy status. Between‐study variance (*τ* ^2^) varied across subgroups and was the highest among studies involving humans, male host, crossbred animals, RT‐PCR‐based diagnostics, animals without a reported abortion history, and non‐pregnant subjects, indicating substantial residual heterogeneity within these categories.

**FIGURE 2:**
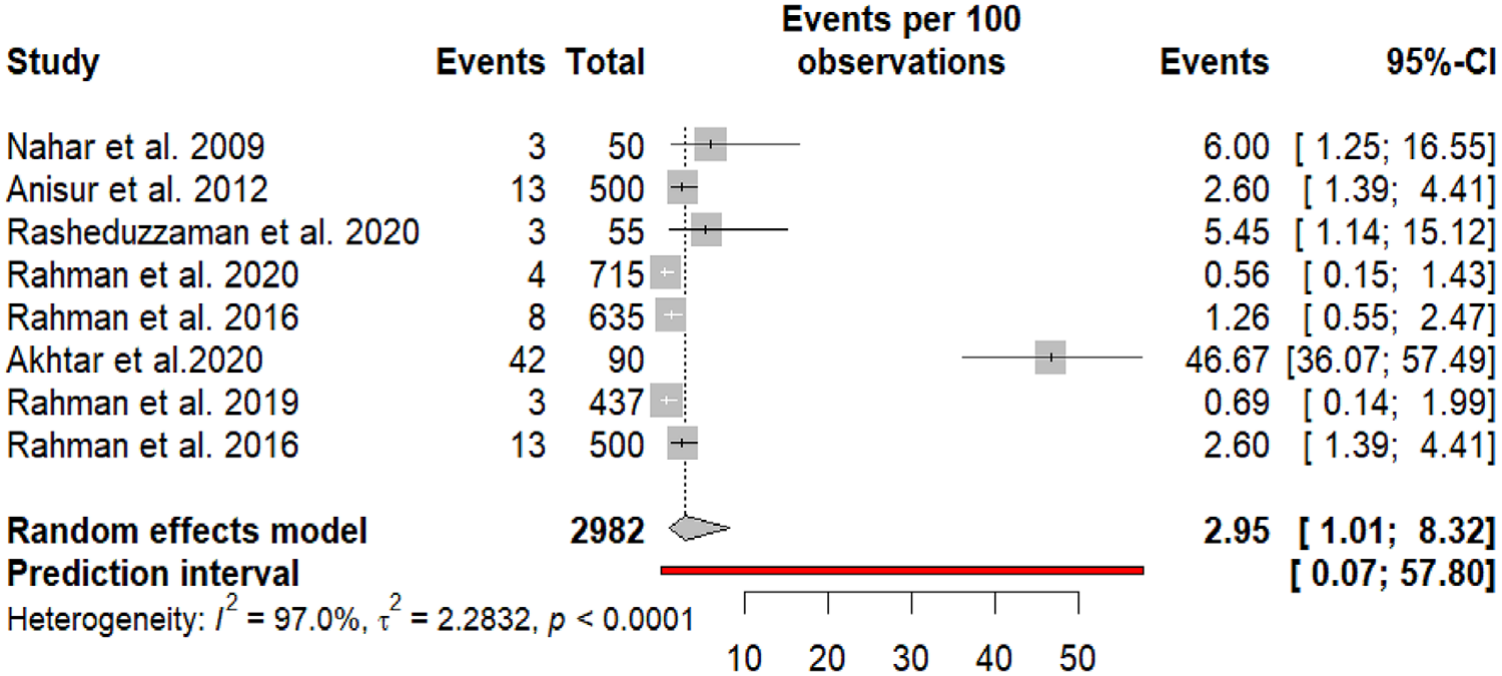
Study‐level and pooled prevalence estimates of human *Brucella* in Bangladesh.

Overall, the pooled prevalence of *Brucella* detection across human and animal hosts was 3.75% (95% confidence interval [CI]: 3.07–4.56), with substantial heterogeneity (*I* ^2^ = 89.5%, *p* < 0.001). Species‐specific subgroup analysis showed the highest pooled *Brucella* detection in dogs (7.49%, 95% CI: 3.23–16.43). Lower pooled estimates were observed for humans (2.941%, 95% CI: 1.00–8.31 (Figure 2), and horses (1.78%, 95% CI: 0.44–6.85) (Table 4). Breed‐specific analysis indicated higher detection in crossbred animals (5.19%, 95% CI: 3.54–7.55). *Brucella* detection estimates varied across diagnostic methods, with higher pooled estimates observed in studies using the *Brucella* antigen kit (5.13%, 95% CI: 3.15–8.27), followed by RBT (4.07%, 95% CI: 3.30–4.99), and ELISA (3.35%, 95% CI: 2.31–4.82). These differences likely reflect variation in diagnostic sensitivity, study populations, and sampling strategies rather than test performance alone. Sex and reproductive‐status‐specific analyses showed higher prevalence among female animals (4.15%, 95% CI: 3.46–5.46), pregnant animals (8.80%, 95% CI: 6.64–11.91), and animals with a history of abortion (30.13%, 95% CI: 20.60–41.74) (Table 4).

### 3.3 Factors Associated With *Brucella* Detection

Univariable meta‐regression analyses were conducted to explore study‐level factors associated with *Brucella* detection (Table 5). Among all evaluated variables, abortion history showed a strong association with *Brucella* detection, with animals having a history of abortion exhibiting markedly higher odds of *Brucella* detection compared with those without abortion history (OR = 10.09, 95% CI: 4.64–21.93). Physiological status also demonstrated a statistically significant association at both the between‐group and within‐group levels. Pregnant animals had higher odds of *Brucella* detection compared with non‐pregnant animals (OR = 1.71, 95% CI: 1.03–2.84). In contrast, species, sex, breed, diagnostic test, and sample type did not show statistically significant between‐group differences in *Brucella* prevalence.Although some species (e.g., dogs, pigs, and buffalo) showed higher estimated odds of Brucella detection compare to horses. No significant differences were observed across diagnostic test types or sample type.

**TABLE 4:** Pooled prevalence of *Brucella* detection in humans and animals.

Category	Subgroup	No. of study	No. of sample	No. of positive	Pooled prevalence (%) (95% CI)	*Q*	*I* ^2^ (%)	Tau square	*p* value (within group)	*p* value (between group)	Egger's test
Overall		54	24,966	1125	3.75 (3.07–4.56)	678.06	89.5	0.62	<0.001		<0.001
Species	Cattle	32	13,343	571	3.67 (2.80–4.77)	283.65	89.1	0.51	<0.001	0.324	0.001
	Goat	12	4166	254	3.86 (2.78–5.34)	59.58	81.5	0.2	<0.001		<0.001
	Sheep	10	3610	164	3.95 (2.57–6.02)	74.27	87.9	0.37	<0.001		0.136
	Buffalo	6	568	32	5.55 (3.53–8.62)	9.82	49.1	0.13	0.080		0.041
	Horse	1	112	2	1.78 (0.44–6.85)	0	—	—	—		—
	Human	8	2982	89	2.94 (1.00–8.31)	230.69	97	2.28	<0.001		0.112
	Dog	2	80	6	7.49 (3.22–16.43)	2.11	52.6	0.04	0.146		—
	Pig	1	105	7	6.66 (3.21–13.32)	0	—	—	—		—
Sex	Male	22	12,638	112	3.56 (2.05–6.11)	171.92	87.8		<0.001	0.501	0.001
	Female	38	10,291	456	4.35 (3.46–5.46)	285.64	87	0.41	<0.001		0.009
Breed	Exotic	2	599	17	2.83 (1.77–4.51)	2.75	63.7	0	0.097	0.142	—
	Local	12	2110	97	4.16 (2.71–6.31)	55.84	82.1	0.35	<0.001		0.108
	Cross	15	6397	330	5.19 (3.54–7.55)	167.18	91.6	0.52	<0.001		0.290
Test	RBT	43	20,351	933	4.07 (3.30–4.99)	398.5	89.5	0.4	<0.001	0.135	0.001
	TAT	4	1720	42	2.44 (1.80–3.28)	3.38	11.3	0	0.336		0.386
	MRT	6	3968	102	2.24 (0.86–5.75)	86.33	94.2	1.37	<0.001		0.121
	SAT	13	7690	252	3.29 (2.05–5.22)	149.7	92	0.67	<0.001		0.152
	*Brucella* antigen kit	3	1022	51	5.13 (3.15–8.27)	9.93	79.9	0.13	0.007		0.782
	ELISA	29	12,851	430	3.35 (2.31–4.82)	438.17	93.6	0.94	<0.001		0.214
	Conventional PCR	4	1624	25	3.1 (0.91–9.94)	25.68	88.3	1.25	<0.001		0.198
	RT‐PCR	6	2480	57	3.33 (0.89–11.61)	100.9	95	2.56	<0.001		0.577
Sample	Blood	51	19,696	830	3.99 (3.09–5.13)	696.59	92.8	0.79	<0.001	0.801	0.006
	Milk	6	3681	143	4.30 (2.48–7.33)	71.94	93.1	0.45	<0.001		0.253
Abortion status	Abortion	22	551	155	30.13 (20.60–41.74)	59.7	64.8	1.05	<0.001	<0.001	0.353
	No abortion	15	4435	219	4.08 (2.30–7.13)	271.86	94.9	1.2	<0.001		0.001
Physiological status	Pregnant	17	1032	98	8.80 (6.44–11.91)	37.92	57.8	0.29	0.001	0.031	0.017
	Non‐pregnant	17	1958	115	5.26 (3.69–7.45)	52.4	69.5	0.36	<0.001		0.010

**TABLE 5:** Publication bias assessment with Egger's test.

Standard effect	Coefficient	*t*‐value	*p* value	95% Cl
Slope	2.25	18.94	0.000	2–2.49
Bias	−0.56	−4.52	0.000	−0.80 to −0.31

### 3.4 Publication Bias Assessment

**FIGURE 3:**
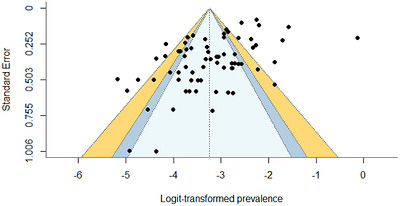
Funnel plot assessing publication bias in the meta‐analysis of *Brucella* prevalence in Bangladesh.

The funnel plot of logit‐transformed prevalence against standard error showed an overall triangular distribution, with most studies falling within the expected pseudo 95% confidence limits; however, some asymmetry was visually apparent, particularly among smaller studies (Figure 3). Egger's test provided statistical evidence of small‐study effects, with a significant intercept (bias coefficient = −0.56, 95% CI: −0.80 to −0.31; *p* < 0.001). The significant slope coefficient (2.25, 95% CI: 2.00 to 2.49; *p* < 0.001) further indicates a strong relationship between study precision and reported effect sizes. Together, these findings suggest the presence of publication bias or other small‐study effects in the included literature.

### 3.5 Spatial Distribution of *Brucella* Detection

**FIGURE 4:**
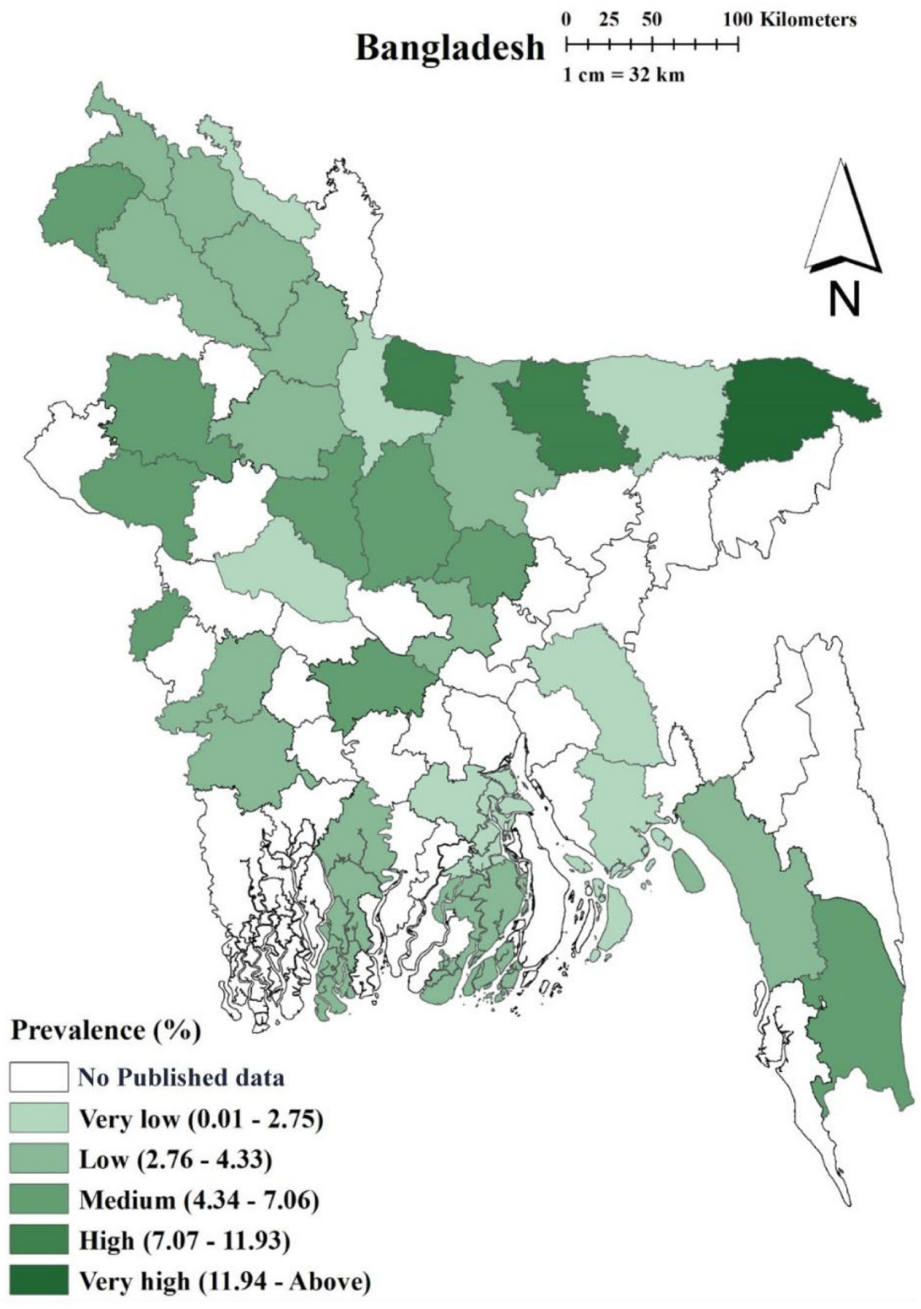
Map showing pooled prevalence of *Brucella* in different districts of Bangladesh.

District‐level choropleth mapping revealed marked spatial heterogeneity in *Brucella* detection across Bangladesh (Figure 4). Higher *Brucella* detection clusters were predominantly observed in the northeastern and northern regions of the country. Sylhet district exhibited the highest reported *Brucella* detection (31.88%), followed by Sherpur (11.83%) and Netrokona (11.11%), placing these districts within the very high prevalence category. In contrast, several districts demonstrated comparatively low prevalence levels, including Sunamganj (1.90%) and Lalmonirhat (2.14%), which fell within the very low prevalence category. Many districts, particularly in central and southeastern Bangladesh, showed either low‐to‐medium detection or lacked sufficient data for estimation. Overall, the spatial patterns indicate substantial geographic variation in *Brucella* detection across Bangladesh.

### 3.6 Trend Analysis and Forecasting

**FIGURE 5:**
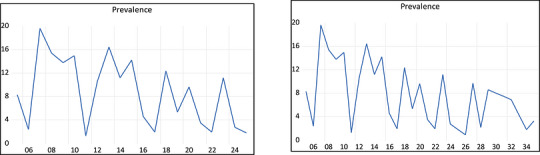
Temporal trend (2005–2024) and forecast (2025–2035) of *Brucella* detection in goats of Bangladesh.

The temporal analysis of goat *Brucella* detection from 2005 to 2024 showed substantial interannual variability, with no evidence of a stable monotonic trend.The period was selected because no comparable published data were available prior to 2005. *Brucella* detection fluctuated markedly over time, characterized by intermittent peaks and troughs, indicating an unstable disease pattern rather than a consistent increase or decrease (Figure 5). Forecasting of goat *Brucella* detection for the period 2025–2035 likewise suggested continued variability, with alternating years of low and relatively high predicted prevalence (Figure 5). The lowest forecasted prevalence was observed in 2026 (point estimate: 0.95%), whereas pronounced peaks were projected for 2027 (9.70%), 2029 (8.59%), and 2030 (8.04%). From 2031 onward, the model indicated a gradual decline in detection, reaching lower predicted levels by 2034 (1.82%), followed by a modest increase in 2035 (3.27%) (Table S1).

**FIGURE 6:**
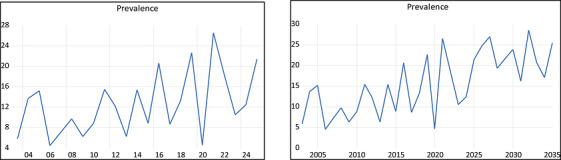
Temporal trend (2003–2024) and forecast (2025–2035) of *Brucella* detection in cattle.

In contrast to goats, time‐series analysis of cattle *Brucella* detection from 2003 to 2024 revealed an overall increasing tendency, although substantial interannual variability was evident throughout the study period (Figure 5). Reported prevalence fluctuated markedly between years, with the lowest observed *Brucella* detection occurring in 2006 (approximately 4%) and a pronounced peak in 2022 (around 25%), indicating an unstable but upward‐leaning disease pattern. Forecasts for the period 2025–2035 suggested that cattle *Brucella* detection is likely to remain relatively high and variable (Figure 6; Table S1). Point estimates indicated prevalence values largely ranging between ∼16% and ∼28%, with the highest projected prevalence occurring around 2032, exceeding 25%. Although short‐term declines were projected in some years, the overall forecast did not indicate a sustained reduction, instead suggesting persistent transmission with notable fluctuations. The model demonstrated a good fit to the observed data, as indicated by low MAE and RMSE values. For cattle, the MAE and RMSE were 0.019 and 0.074, respectively, while for goats, the corresponding values were 0.145 and 0.102 (Table 6). These relatively small error estimates suggest that the model adequately captured the underlying temporal patterns and is suitable for short‐ to medium‐term forecasting. Nevertheless, given the observed temporal variability, forecasted estimates should be interpreted with appropriate caution.

**TABLE 6:** Summarization of the MAE and RMSE for the goat and cattle models.

Variable	MAE	RMSE
Goat	0.145	0.102
Cattle	0.019	0.074

## 4 Discussion

This systematic review and meta‐analysis synthesized savailable articles on *Brucella* detection across human and animal hosts in Bangladesh, providing a quantitative yet context‐dependent estimate of detection at 3.75% (95% CI: 3.07–4.56). Importantly, this estimate should not be interpreted as a direct measure of disease burden, but rather as a structured summary of heterogeneous evidence derived from diverse diagnostic and study designs. A key finding is that *Brucella* detection operates within a multi‐host system, with relatively comparable estimates across major livestock species. This pattern suggests widespread exposure across host communities rather than species‐specific susceptibility, consistent with ecological dynamics of endemic zoonoses. This study found a pooled Brucella detection of 3.75% across all species in Bangladesh, which is lower than that reported in some other systematic reviews and meta‐analyses conducted in Iran (Khoshnood et al. 2022) and Africa (Simpson et al. 2021). This difference may be attributed to several factors, including variations in host species, temperature, humidity, test accuracy, and underreporting. Although higher estimates were observed in dogs and lower estimates in horses, these results are constrained by limited sample sizes and wide CIs, reducing inferential strength and precluding strong conclusions about species‐specific differences. Since the current study included very few studies that reported *Brucella* detection in dogs and horses, these findings cannot represent the exact burden of brucellosis in those species. Besides, various livestock species showed persistent *Brucella* detection estimates in Bangladesh, suggesting that *Brucella* spp. exists in the region as a multi‐host infection system rather than being driven by a single dominant species. The relatively comparable prevalence estimates across cattle, goats, sheep, buffalo, and pigs indicate widespread exposure rather than species‐specific susceptibility. Additionally, the reported species‐specific seroprevalence of brucellosis in Bangladesh is lower than that reported in other studies conducted in India (Khoshnood et al. 2022), Pakistan (Simpson et al. 2021), and Nepal (Acharya et al. 2016). Although these countries share a similar ecological and socio‐economic setting, this discrepancy may be attributable to underreporting.

Study heterogeneity emerged as a dominant driver of variation, particularly with respect to diagnostic approach. Serological assays (e.g., ELISA, RBT) capture historical exposure, whereas PCR‐based methods detect current presence of bacterial DNA, leading to fundamentally different epidemiological interpretations (Dadar et al. 2025). Consequently, variation observed across studies is more plausibly attributable to differences in management practices, herd structure, and surveillance intensity than to intrinsic host biological susceptibility. Accordingly, differences in reported prevalence across studies likely reflect diagnostic context rather than true variation in transmission intensity, reinforcing that pooled estimates are inherently method‐dependent. This distinction is critical in endemic systems, where chronic exposure and repeated circulation can inflate serological signals relative to active infection.

Breed and sex specific differences were observed but were not statistically significant in meta‐regression. Similarly, higher prevalence in females and crossbred animals was observed, consistent with previous studies (Tadesse 2016; Hassan et al. 2014; Mcdermott et al. 2013; Dadar et al. 2025). Diagnostic method was a major source of heterogeneity. Higher estimates from serological assays compared to PCR reflect the detection of past exposure rather than active infection. This discrepancy is well documented in endemic settings (Freire et al. 2024; Yagupsky et al. 2019) and highlights that prevalence estimates are strongly influenced by diagnostic approach. Among evaluated variables, abortion history and physiological status emerged as the consistent correlate of *Brucella* positivity, supporting well‐established biological mechanisms linking *Brucella* spp. to reproductive tissues (M.J. Corbel et al. 2006; Alsaif et al. 2018). However, this association should be interpreted cautiously as an epidemiological linkage rather than causal evidence, as abortion both results from infection and increases the likelihood of detection through targeted testing. In contrast, variables such as sex and breed did not retain significance after accounting for study‐level heterogeneity, suggesting that these factors likely act as proxies for exposure intensity, management practices, or sampling bias rather than independent determinants of infection (Tadesse 2016; Alsaif et al. 2018).

Spatial and temporal patterns further illustrate the limitations of inference from heterogeneous literature. Higher reported prevalence in northern and northeastern regions likely reflects greater livestock density and research activity, rather than true geographic hotspots of transmission (Musallam et al. 2016). Similarly, the observed increase in cattle and decrease in goats over time should be interpreted with caution, as these trends are derived from study‐reported data rather than standardized surveillance systems. Such apparent trends may therefore arise from shifting diagnostic practices, study focus, and sampling strategies, rather than underlying epidemiological change (Munsi et al. 2021; Stanley and Jarrell 1989).

Overall, these findings demonstrate that *Brucella* prevalence estimates are highly context‐dependent, shaped by diagnostic methods, study design, and sampling structure. Univariable associations should therefore be interpreted as indicators of exposure structure rather than definitive risk factors, and pooled estimates should be viewed as summaries of heterogeneous evidence rather than precise population‐level metrics. This study provides one of the first integrated syntheses across human and multiple animal hosts in Bangladesh, offering a baseline for future research and policy. Moving forward, progress in understanding *Brucella* epidemiology will depend on standardized diagnostics, improved geographic coverage, and longitudinal surveillance capable of distinguishing true transmission dynamics from methodological artifacts.

## 5 Conclusion

This study shows that *Brucella* detection remains present across human and animal hosts in Bangladesh, with a pooled prevalence estimate of 3.75%. Subgroup analyses suggest higher detection in dogs, females, and crossbred animals; however, these differences were not statistically significant. Among study‐level variables, abortion history was the only correlate of *Brucella* positivity. Temporal trends showed an increase in cattle and a decrease in goats. The spatial patterns varied across studies, likely reflecting differences in study design, diagnostics, and coverage rather than true epidemiological trends. Overall, these findings highlight the need for cautious interpretation of pooled estimates and emphasize the importance of standardized methods in future *Brucella* research.

### Recommendations

Future *Brucella* studies in Bangladesh should prioritize standardized diagnostic and reporting frameworks to improve comparability and reduce methodological heterogeneity in pooled analyses. Transparent reporting of reproductive history, particularly abortion status, should be emphasized, given its consistent association with *Brucella* positivity across studies. Improved geographic coverage and more balanced host representation are needed to minimize spatial and species‐level biases and strengthen inference on prevalence patterns. The interpretation of prevalence estimates should explicitly account for diagnostic context, avoiding direct comparisons across fundamentally different detection methods. Where data permit, future studies should consider integrated analyses across animal and human hosts to better characterize shared exposure pathways and risk structures. This gap reflects surveillance, diagnostic, and study design limitations rather than biological differences. Finally, time‐series and forecasting analyses should be treated as descriptive summaries of published studies rather than proxies for structured surveillance, given their sensitivity to heterogeneous study inputs. These recommendations are intended to enhance the quality, consistency, and interpretability of future *Brucella* research in Bangladesh, without extending beyond the evidence generated in this study.

## Author Contributions

**Radwan Raquib**: conceptualization, methodology, formal analysis, writing – review & editing. **Farhaj Ahammed Arnob**: investigation, validation, data curation. **Riyadh Hossain**: conceptualization, methodology, formal analysis, writing – review & editing. **Ahsan Raquib**: formal analysis, visualization, investigation, writing – original draft. **Tajul Islam Mamun**: methodology, supervision, writing – original draft, writing – review & editing. All authors read and approved of the final manuscript.

## Funding

The authors have nothing to report.

## Ethics Statement

Ethical approval was not required for this study, as it involved secondary data analysis and did not include live animals and human subjects.

## Supporting information




**Supporting file 1**: vms371005‐sup‐0001‐SuppMat.zip

## Data Availability

None.
