# Supplementary material for: Prevalence, Associated Factors, and Temporal Trends of Brucella Detection Across Human and Animal Hosts in Bangladesh: A 25‐Year Meta‐Analysis
Source: Vet Med Sci. 2026 May 28;12(4):e71005. doi: 10.1002/vms3.71005 (PMC13239763; doi:10.1002/vms3.71005)
Supplement: Supplementary file 1 — Supporting file 1: vms371005‐sup‐0001‐SuppMat.zip [file VMS3-12-e71005-s001.zip › supplementary materials.docx]

**
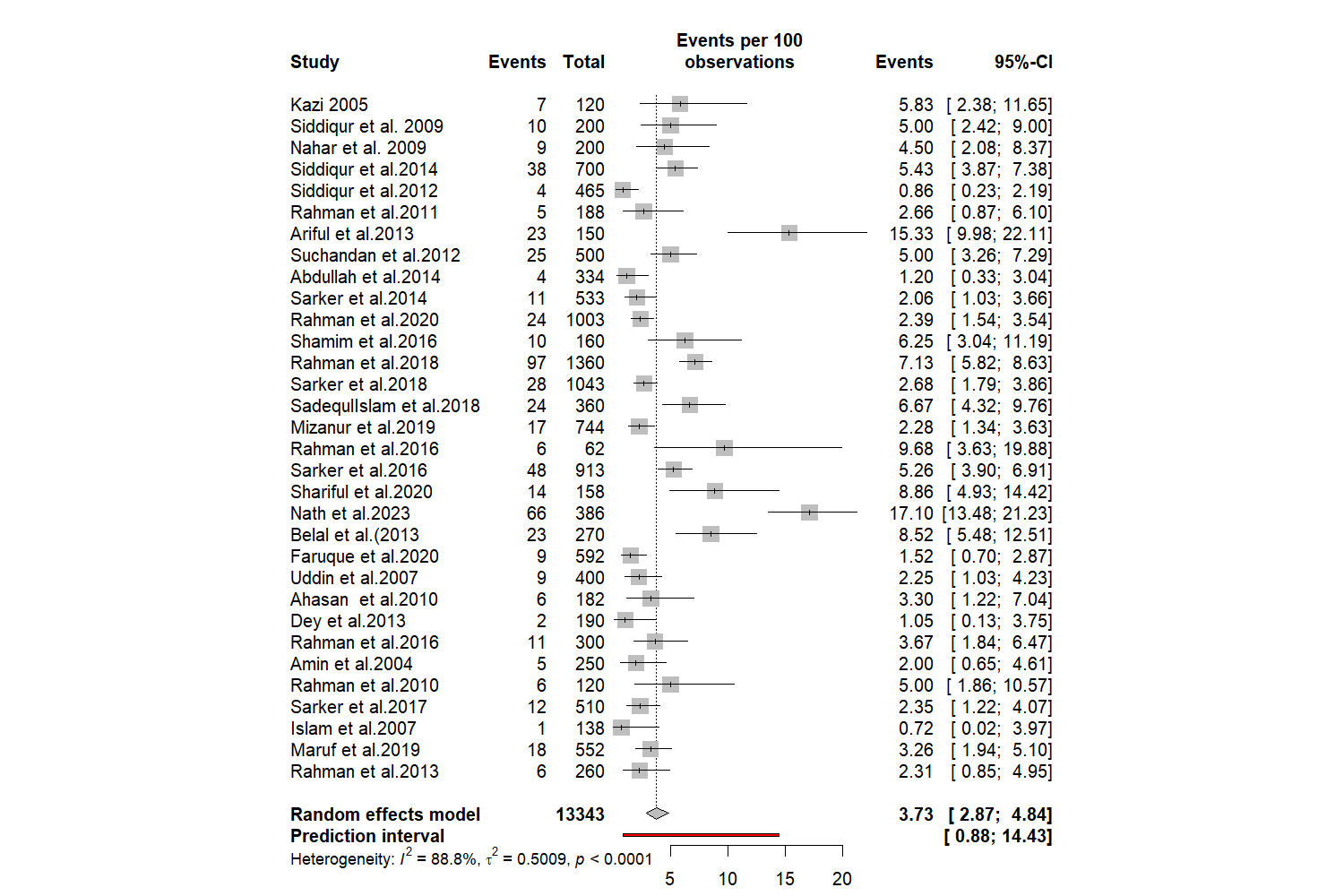
**

**Figure 01:** Forest plot showing study-level and pooled prevalence estimates of bovine brucella in Bangladesh.

**Table 01:** Forecasting values for goat and cattle *brucella* from 2025 to 2035

| **Time** | **Goat** | | | **Cattle** | | |
| --- | --- | --- | --- | --- | --- | --- |
| Year | Point forecast | Lower  CI(95%) | Upper  CI(95%) | Point forecast | Lower  CI(95%) | Upper  CI(95%) |
| 2025 | 1.806084 | 1.2549 | 2.4568 | 21.39299 | 18.3245 | 24.4587 |
| 2026 | 0.952282 | 0.3654 | 3.2657 | 24.67261 | 22.4258 | 26.1258 |
| 2027 | 9.69848 | 8.0125 | 11.6578 | 26.97295 | 24.7854 | 29.1876 |
| 2028 | 2.144678 | 1.4568 | 3.2536 | 19.2733 | 17.7546 | 22.4532 |
| 2029 | 8.590877 | 6.2358 | 10.6528 | 21.57365 | 17.1245 | 22.3547 |
| 2030 | 8.037075 | 5.2658 | 11.1254 | 23.87399 | 20.1458 | 25.6356 |
| 2031 | 7.483273 | 5.2363 | 10.2489 | 16.17434 | 11.4532 | 21.2536 |
| 2032 | 6.929472 | 4.7824 | 7.6549 | 28.47468 | 26.4521 | 31.4521 |
| 2033 | 4.37567 | 3.4587 | 6.2459 | 20.77503 | 17.2736 | 22.6324 |
| 2034 | 1.821868 | 0.9587 | 3.2164 | 17.07538 | 15.7634 | 20.3654 |
| 2035 | 3.268067 | 1.5368 | 4.1258 | 25.37572 | 23.2136 | 28.4521 |
